# Exposure to multiple career pathways by biomedical doctoral students at a public research university

**DOI:** 10.1371/journal.pone.0199720

**Published:** 2018-06-22

**Authors:** Ambika Mathur, Christine S. Chow, Andrew L. Feig, Heidi Kenaga, Judith A. Moldenhauer, Nisansala S. Muthunayake, Mathew L. Ouellett, Laura E. Pence, Victoria Straub

**Affiliations:** 1 Graduate School, Wayne State University, Detroit, Michigan, United States of America; 2 Department of Chemistry, Wayne State University, Detroit, Michigan, United States of America; 3 Department of Art and Art History, Wayne State University, Detroit, Michigan, United States of America; 4 Office for Teaching and Learning, Wayne State University, Detroit, Michigan, United States of America; 5 Department of Chemistry, University of Hartford, Hartford, Connecticut, United States of America; 6 SPEC Associates, Detroit, Michigan, United States of America; Indiana University, UNITED STATES

## Abstract

The Broadening Experiences in Scientific Experiences (BEST) program at Wayne State University was designed to increase doctoral students’ awareness of multiple employment sectors beyond academia, improve their knowledge of transferable skills required to succeed in any career path, provide opportunities to explore diverse career paths, and gain in-depth knowledge about those paths using experiential learning opportunities. We devised a three-phase program that ranged from providing students with a broad introduction to multiple career opportunities to immersive experiential learning in a specific career sector. Importantly, program content was developed and delivered by alumni and industry experts in five employment sectors–business/industry, communication, government, law/regulatory affairs, and undergraduate/PUI teaching–in partnership with WSU faculty. This article provides data on two notable outcomes: doctoral students participate equally in BEST activities regardless of gender, race, and citizenship status, and student participation in BEST activities did not correlate with lower GRE ratings, lower GPA, or increased time-to-degree. Further, a “halo” effect of the program is evidenced by participation of students from all disciplines, not just the biomedical sciences. Centralizing BEST activities within the Graduate School will allow faculty and individual programs to save resources and time.

## Introduction

The graduate training community has traditionally focused on preparing doctoral students for jobs in academia. Recent reports on career outcomes show, however, that more than half of U.S. biomedical doctoral recipients pursue careers beyond academia [1–3]. The graduate and scientific training communities and federal funding agencies are now beginning to accept these multiple career pathways as successful doctoral training outcomes [4–6]. It is therefore important to ensure that academic institutions and individual doctoral programs understand these career trajectories and shift current training paradigms to provide students with the appropriate resources required for success in these sectors [7–10]. However, doctoral programs often have little experience in providing their students with access to these types of opportunities. Further, trainees need to recognize how their skills are transferable across careers [8–14]. In response, in 2013 the National Institutes of Health (NIH) Common Fund instituted a Broadening Experiences in Scientific Training (BEST) grant, with the goal of assisting academic institutions to provide career exploration and professional development to biomedical doctoral and postdoctoral trainees in preparation for careers beyond academia [15]. Wayne State University (WSU), a major comprehensive research institution located in Detroit, was the recipient of one of these 5-year, nonrenewable grants.

At WSU, the Graduate School awards all Ph.D. degrees and oversees approximately 1,500 Ph.D. students in all disciplines, including 400 students in 15 biomedical programs. A recent census of its 3,000 doctoral alumni who graduated from WSU in the period from 1999–2014 showed that, mirroring the national trend, our biomedical doctoral alumni work in a variety of employment sectors, including industry/business (31%), academia (tenure/tenure-track, 29%), undergraduate teaching (PUIs, 13%), government and law/regulatory sectors (5%), science communication (<1%), and ongoing training (such as postdoctoral positions, 16%) [16]. Additionally, surveys associated with this census reveal that our alumni share the sentiments expressed in national reports–they are extremely satisfied with the research training at WSU, but they also state that they did not receive adequate information about careers outside academia or training in professional skills required to succeed in these careers beyond disciplinary training. Catalyzed by the NIH-BEST award, WSU’s program is designed to address these gaps by providing current students with exposure to various career sectors as well as professional development and training in transferable skills that will better prepare them for these careers. Titled WSU BEST, the program offers a core of related professional development activities, including a three-phase career exploration program, additional seminars, and workshops–all designed by professionals in partnership with WSU faculty–which focus on the following career sectors: business, communication, government, law, and teaching. While rooted in biomedical doctoral education, the WSU BEST program is open to all doctoral students interested in pursuing careers at the intersection of science and other disciplines. WSU BEST also mandated completion of an Individual Development Plan (IDP) for all doctoral students.

There is a perception in the biomedical training community that women and students from underrepresented backgrounds pursue careers in nonacademic sectors in greater percentages than their well-represented counterparts [17–26] and therefore by extension participate more frequently in programming aimed at acquiring transferable skills for success in these sectors. Similarly, there may be a perception that students involved in professional development activities have lower GREs, lower GPAs, and longer time-to-degree rates than nonparticipating students [27, 28]. Therefore, we examined and compared the characteristics (gender, race, U.S. citizenship status, GRE scores, GPA at the time of completion, and time-to-degree completion) of program participants with students who did not participate.

In this article, we share outcomes from WSU BEST programming on student knowledge about multiple careers and transferable skills required for success in these careers, as well as the characteristics and correlation with GRE, GPA, and time-to-degree completion of students participating in this program.

## Materials and methods

### Ethics statement

All research conducted in this program was approved by WSU’s Institutional Review Board on the Use of Human Subjects, IRB#094013B3E.

### Demographic data collection

Demographic information for participants and nonparticipants was obtained from WSU and Graduate School records. Departmental affiliations of participants were obtained from registration and survey records. All data are reported in aggregate or with identifiable information removed.

### Participants

Each year WSU typically enrolls 60 to 70 new students into its 15 biomedical doctoral programs with a total of about 400 biomedical doctoral students. Typically, students participate in the WSU BEST program after completion of their qualifying exams and achieving candidacy (generally in Year 3 of the 5.5-year average time-to-degree completion), although they are encouraged to start their career explorations at any time during their doctoral training. At WSU, the Graduate School oversees all Ph.D. programs and therefore leads the BEST initiative centrally in partnership with faculty in the individual doctoral programs, leadership of the colleges, professionals in industry, and the WSU Office of Teaching & Learning. To be inclusive, the Graduate School invites all 1,500 doctoral students, including those in departments not traditionally associated with the biomedical discipline, to participate in BEST programming. We also encourage participation from master’s students, postdoctoral trainees, and faculty, as well as students from area institutions, although only outcomes of WSU doctoral trainees from the past three years are reported here.

### Program description

We designed WSU BEST to strategically prepare biomedical doctoral students for careers in the sectors identified by our alumni: business/industry, communication, government, law/regulatory affairs, and undergraduate/PUI teaching. These sectors are also critical to the 21^st^ century economy. WSU BEST’s model of biomedical career exposure comprises career planning and preparation (professional development activities). All incoming and current doctoral students are invited to attend WSU BEST’s Orientation Session dedicated to career planning, choices, and guidance. In addition, all Ph.D. students are required to complete an initial IDP by the end of their first year. In addition, they must update it annually to reflect potential changes in their career goals as well as document progress made toward developing critical skills required for career success. The IDP is an invaluable tool in sparking conversations between doctoral trainees and their research mentors during the first semester about their long-term career interests.

### Three-phase program

WSU BEST provides students with information about various careers via a three-phase process, designed to be taken successively. Phase I is open to all doctoral students interested in career exploration; Phase II delivers more detailed information for those who decide they want in-depth learning about one or more of the specific career(s); and Phase III offers hands-on experiential learning to a select number of students interested in immersive experiences in any one career sector.

#### Phase I: Exploratory seminars (Introduction to Careers)

Participation in career-path exploration modules is open to all students and encourages them to think broadly about career options and trajectories. Students can elect to attend one or more 90-minute modules, each involving a panel discussion highlighting career opportunities in that particular sector and providing knowledge of the expectations and skillsets of a professional in that field. Students gain a sense of whether a particular area is of sufficient interest that they want to pursue it further. Seminar presenters include an alumnus or program partners currently working in the specific career sector and a faculty member who facilitates the session. In the first year of the program, all Phase I sessions were videotaped and made available to students via the WSU BEST Blackboard courseware and then later publicly on the WSU YouTube channel (each Phase I video averages a hit rate of 60 times per year).

#### Phase II: Interactive workshops (Career Preparation)

These full-day workshops provide a deeper experience of each career path than the initial Phase I exposure and are open to students who have completed Phase I or viewed modules on Blackboard. Each workshop includes a variety of activities, such as presentations, discussions, interactive projects within mock interdisciplinary teams addressing a typical task within that career path, and conclude with group presentations about the results of their assigned task and collaboration. Working in conjunction with faculty, alumni and partners in each specific career area develop module content and learning outcomes, lead the activities, and provide feedback to participants.

#### Phase III: Career explorations (In-depth Experiential Learning)

The culmination of the student experience in WSU BEST is open to a smaller group of students (10 or so each year, selected via a competitive process) who want more extensive training involving hands-on experiential learning with a partner organization. The duration and structure for each experience is established in collaboration with the partner, typically totaling 160 hours over the summer months. Application requirements include a brief essay, transcript, up-to-date IDP, and approval by the research mentor and the department’s Director of Graduate Studies. Upon completion of Phase III, all participants are required to submit a report about their experience.

#### Engagement of alumni and industry partners in BEST programming

From 2014–17, the BEST program engaged 46 industry, government, and community partners, along with 21 WSU faculty and staff, in developing its Phase I seminars and Phase II workshops. Approximately half of these presenters were WSU alumni who had successfully pursued nonacademic career trajectories. The BEST program partnered with the WSU Office of Teaching & Learning to create a model that focused on outcomes specific to the individual module topics. All module exercises involved active-learning components for the students. Panelists for the Phase I seminars presented narratives about their individual career paths, followed by responses to a set of questions posed by a BEST-affiliated faculty facilitator. For the Phase II daylong workshops, presenters introduced exercises and provided individualized direction as needed, and students then completed the exercises in small groups. This allowed students to collaborate with those from diverse disciplines and benefit from the shared expertise of other participants.

#### Graduate and postdoctoral professional development (GPPD) seminars

In coordination with BEST, the WSU Graduate School offers a weekly series of seminars and workshops that cover a range of transferable skills and topics that are of interest to doctoral students as they prepare for their careers. This series, conducted across the academic year, is designed to help students develop and demonstrate core competencies such as communication, ethics, teamwork and collaboration, leadership and professionalism, with more targeted topics such as developing an effective LinkedIn profile page, writing resumes and cover letters, and practicing negotiation skills. Faculty, alumni, and industry professionals are engaged in the design and delivery of these sessions.

#### Tracking student participation and assessing correlation of GRE scores, cumulative GPA at time of graduation, and time-to-degree with participation in BEST activities

We tracked participation of students in IDP completion, the BEST Orientation, the BEST three phases, and other professional development activities (*e*.*g*., GPPD seminars). Demographic information such as gender, race/ethnicity, and citizenship was recorded. GRE scores, cumulative GPA, and time-to-degree completion as well as current employment information of graduates were obtained from WSU official centralized student databases in the Graduate School.

### Program evaluation

The three important outcomes that BEST aimed to achieve as precursors to student career placement included: (1) increased awareness of career options in addition to academia; (2) more access to opportunities, guidance, and support to pursue diverse careers; and (3) greater interest and intent to pursue diverse careers. These outcomes were assessed by measuring students’ perceptions of change [29] using the Retrospective Pretest (RPT) methodology [30–34]. The primary source of data for formative and summative evaluations used to assess these outcomes were surveys (see Supplementary Materials) completed by students at the end of their participation in each Phase I and Phase II seminar/workshop, and after their Phase III experience. Evaluation of student participation in and their perceptions about BEST activities was conducted by SPEC Associates, a third-party nonprofit research and evaluation organization based in Detroit. The major formative evaluation question was: How do students rate the quality of each program component and what reasons do they give for their ratings? The two major summative questions were: (1) Do students report gains in knowledge about nonacademic career opportunities and the skills needed to pursue them, and (2) Do students report changes in interest in nonacademic career opportunities as a result of participation in the program? Each item was rated on a scale of 1 meaning “nothing/not at all” to 5 meaning “a great deal,” with the interim points on the scale left undefined. A second series of questions asked respondents to reflect on their level of the same knowledge or skills prior to participation in the intervention. The difference between the “now” and “then” ratings constituted the measure of change.

### Statistical analysis

The Student’s two-tailed paired test was used to calculate “p” values to determine whether differences between comparison groups were significant. Across all comparisons presented in this report, “p” values equal to or less than 0.05 were considered to be significant differences between comparison groups.

## Results

### Student participation in professional development activities before and after BEST

**Fig 1:**
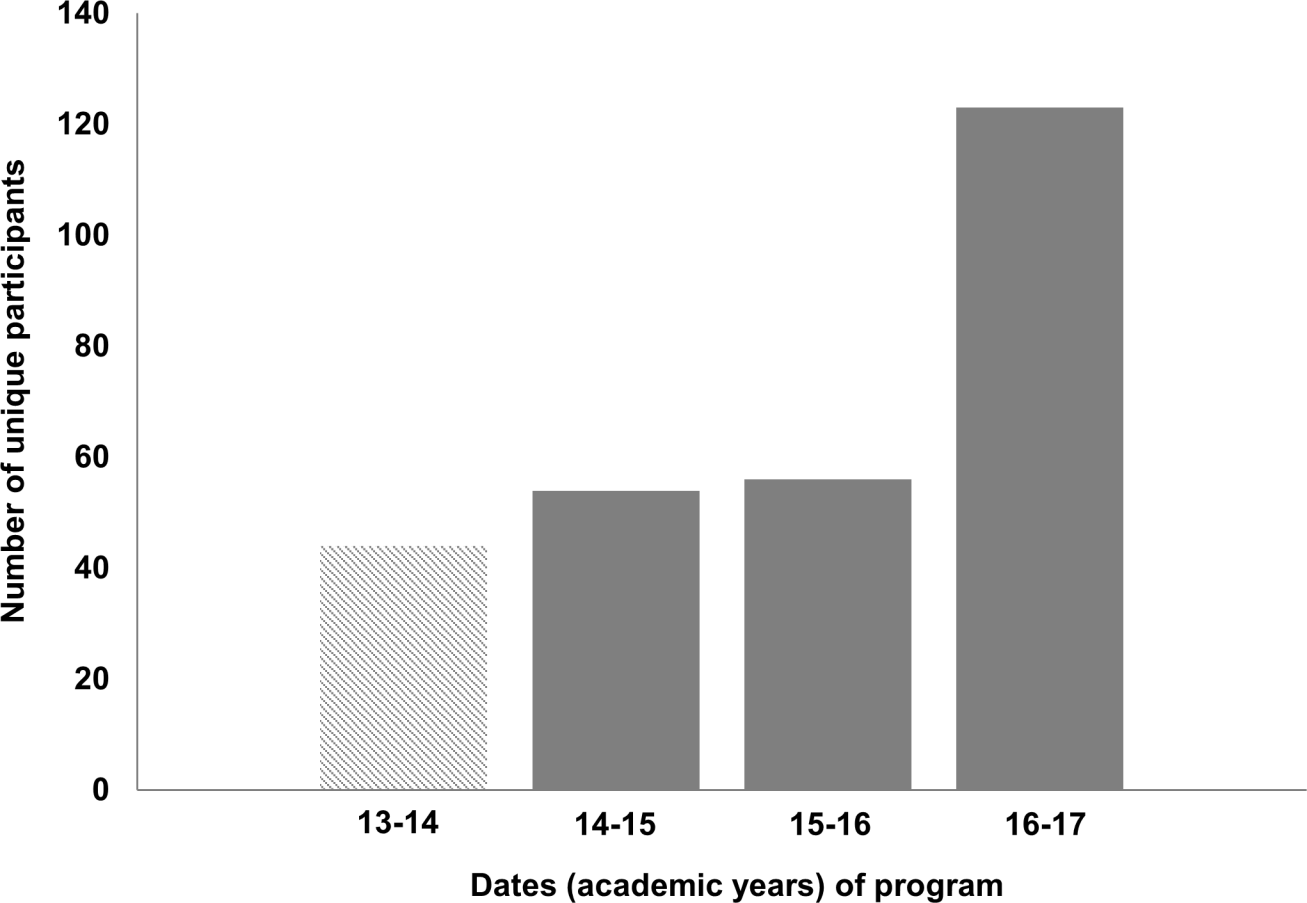
Participation of doctoral students from biomedical departments in BEST events. Pre-BEST vs. BEST participation (unique participants) 2013–17. The striped bars represent participants from 2013–14, the "pre-BEST" period. The solid bars represent BEST participants from 2014–17.

To determine the overall interest in diverse career opportunities provided by the WSU BEST program, we compared doctoral student attendance at professional development activities in the year immediately prior to WSU’s BEST grant award (pre-BEST, 2013–14), with attendance in the years following receipt of the grant (2014–17). Student participation in each phase or professional development event (*e*.*g*., Phase I, II, III, or GPPD seminars) was counted, regardless of the duration (event times range from 1–2 hours for GPPDs and Phase I seminars, 1–2 days for Phase II and GPPD workshops, and 1–160 hours for Phase III experiences). Student participants attended 1–13 events, with an average of 2 events per student. For reporting purposes, we defined each individual as a “unique participant.” We found that attendance in professional development activities increased each year, especially in 2016–17 (2.8-fold higher compared to pre-BEST and 2.2-fold higher than 2015–16) among students in biomedical-related departments (see **Fig 1**).

In 2016–17, a total of 123 unique students participated from biomedical and 46 from non-biomedical departments. Overall participation for the three-year period (2014–17) was 44% of all doctoral students in the biomedical departments. In other words, *nearly half* of the biomedical students participated in career development since establishment of the BEST program. Non-biomedical department student participation ranged from 1 to 10%.

### Demographics of students participating in BEST activities

**Fig 2:**
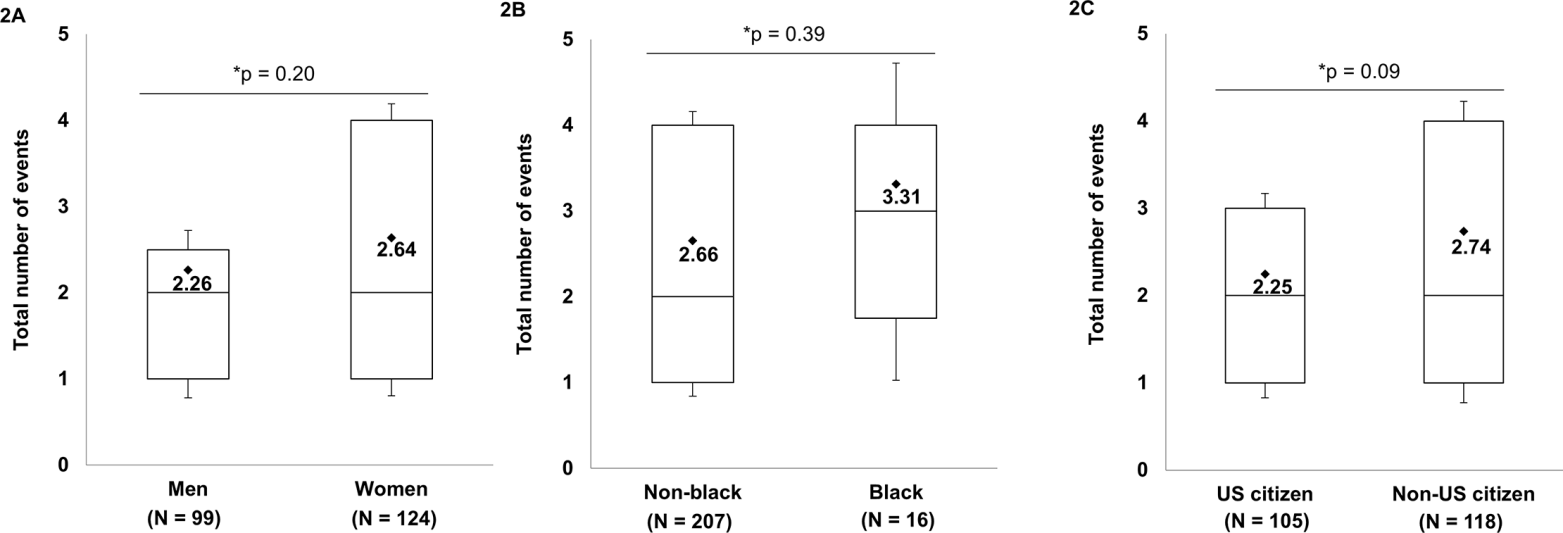
Demographics of doctoral students participating in BEST events. The number of events attended by unique participants from 2014–17 (total N = 223) are displayed by (A) gender, (B) ethnicity, and (C) U.S citizenship status.

Across 2014–17, women comprised slightly more than 50% of biomedical doctoral students; underrepresented minority (URM) students comprised 7%, with Blacks being the predominant URM group and less than 1% of Hispanics, Native Americans and all other groups combined; across the same time period, U.S. citizens/permanent residents comprised 49% of all biomedical doctoral students (see **Fig 2**).

The demographics of students who participated in WSU BEST programming from 2014–17 were as follows: 56% female and 44% male; 7% Black and 93% all other races; 47% U.S. citizen and/or permanent resident and 53% non-U.S. citizen. While not statistically significant, we found that women attended a greater number of BEST activities than men (**Fig 2A**); Black students attended slightly higher numbers of BEST events compared with all other students (**Fig 2B**); and U.S. citizens and permanent residents attended fewer BEST events than non-U.S. citizens (**Fig 2C**).

### Correlation of student GRE, GPA and time-to-degree completion with participation in BEST activities

**Fig 3:**
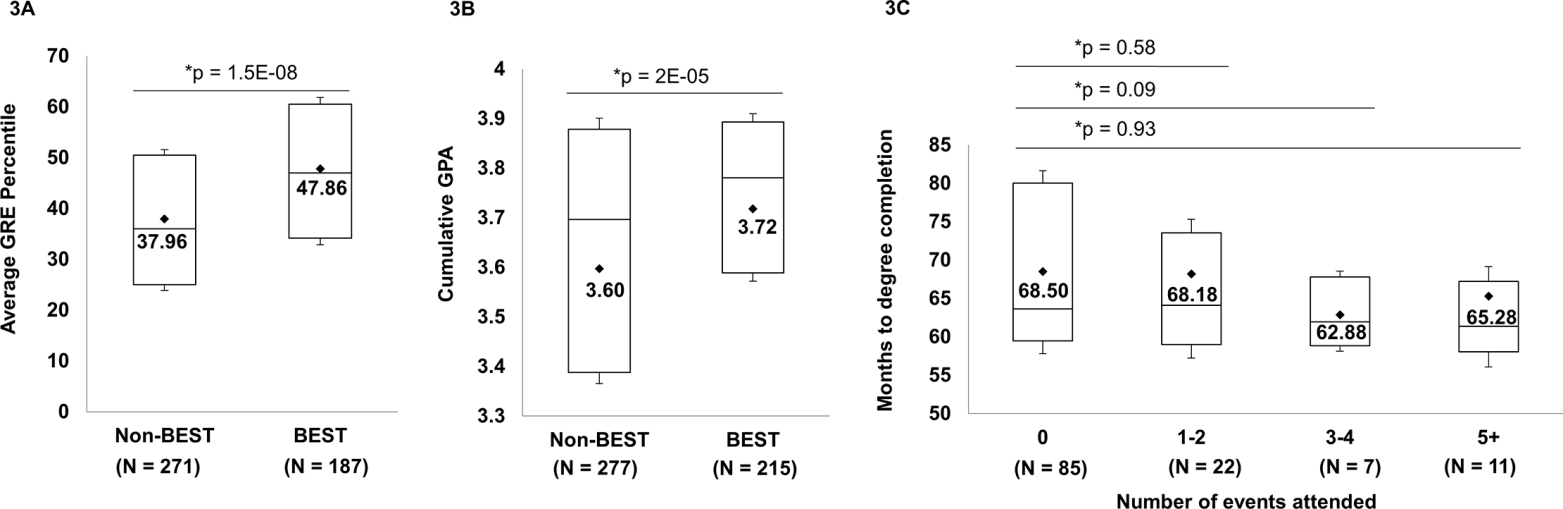
Academic performance of students participating in BEST activities. (A) The average GRE percentile scores for incoming students. Note: some programs do not require GRE scores for admission, so not all BEST participants are included. (B) Cumulative GPAs from 2014–17. Non-BEST students did not participate in any BEST events; BEST students are those who participated in one or more BEST events. (C) Time-to-degree completion for BEST participants who graduated 2014–17. The events include Phases I, II, and III, and GPPD seminars. The duration of each event varied from one hour for GPPDs and Phase I to an average of 160 hours for Phase III participation. In three years, 125 of the unique participants completed their doctoral degrees.

We compared incoming GRE percentile scores of biomedical doctoral students who participated in BEST activities compared with scores of students who did not participate from 2014–17 (see **Fig 3**).

As shown in **Fig 3A**, incoming GRE scores were *significantly higher* among BEST participants compared with nonparticipants. In addition, cumulative GPAs of students who participated in BEST activities were higher than those of nonparticipants (**Fig 3B**) (please note: we are not stating that GPA is an outcome of BEST participation; we are instead examining correlations between graduate school metrics and career development participation). Finally, data in **Fig 3C** show that time-to-degree completion is not affected by participation in BEST activities, even when the number of BEST activities increases from a single event to 5 or more events.

### Impact of WSU BEST program on student knowledge of careers

In Years 1–3 of the program (2014–17), 363 surveys (228 from doctoral students and 135 from nondoctoral attendees, such as postdoctoral scholars and faculty) were completed for Phase I, and 210 surveys (108 from doctoral students) were submitted for Phase II.

**Fig 4:**
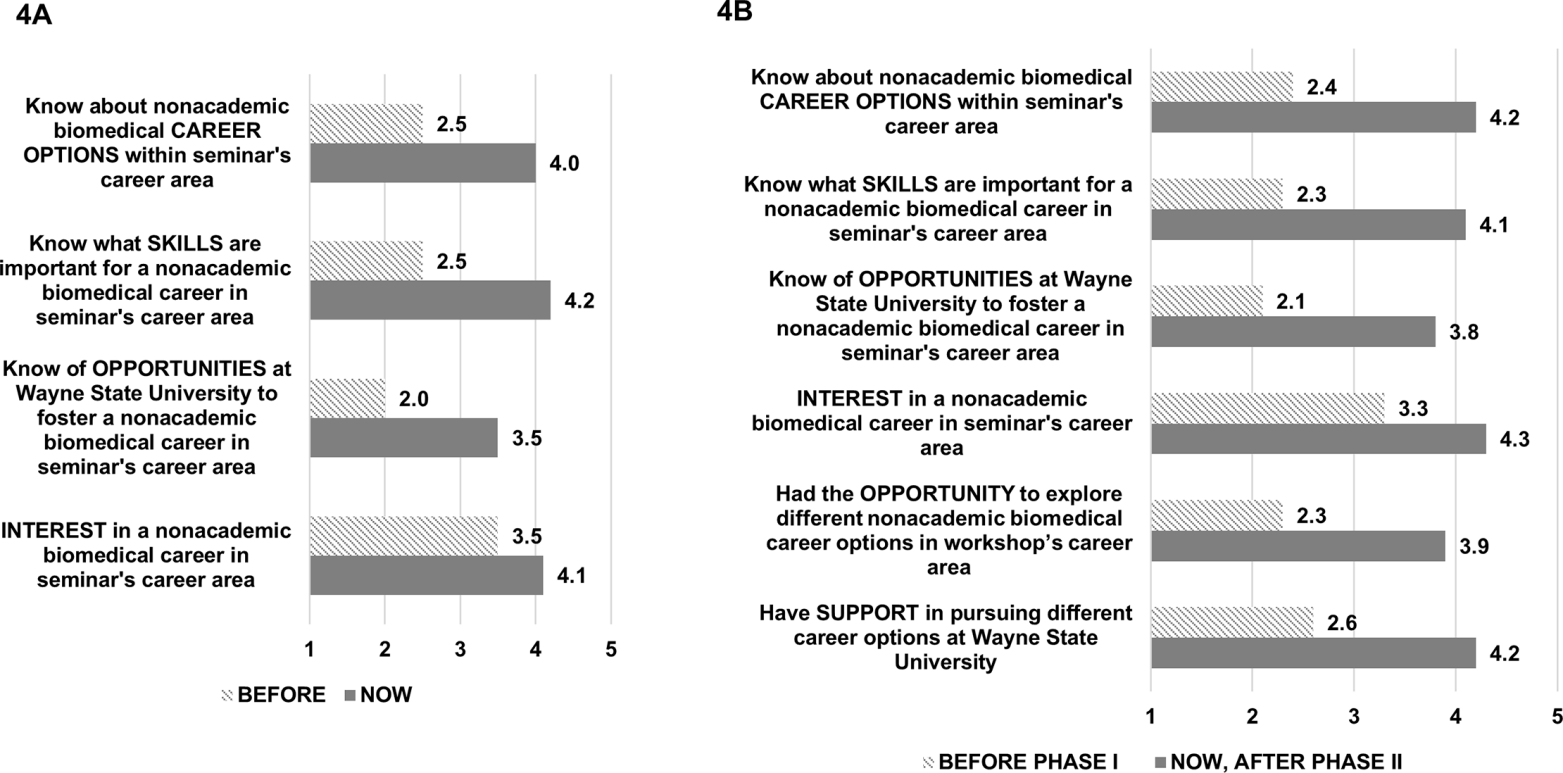
Survey results of doctoral students in BEST target departments. (A) Phase I survey (Years 1–3) (N = 227 surveys), (B) Phase II survey (Years 2–3) (N = 70) results are shown. The scale for both surveys ranges from 1 (nothing/not at all) to 5 (a great deal).

As shown in **Fig 4A**, after Phase I career exploration sessions (Years 1–3 for target departments), there was a statistically significant difference in scores for “before” and “now” ratings in each of the following four areas: (1) know about nonacademic biomedical career options in the specific sector addressed in the seminar; (2) know what skills are important for a nonacademic biomedical career in that sector; (3) know of opportunities at WSU to foster a nonacademic biomedical career in that sector; (4) level of interest in a nonacademic biomedical career in that sector. When comparing biomedical with non-biomedical departments, similar results were obtained with a statistically significant pre-post change for each question (data not shown).

Similar gains in ratings on each of the same four areas plus two additional items were observed for attendees of Phase II (**Fig 4B**). There was a statistically significant increase in scores for “before Phase I” and “now (after Phase II)”: (1) know about nonacademic biomedical career options in that workshop’s career area; (2) know what skills are important for a nonacademic biomedical career in that career area; (3) know of opportunities at WSU to foster a nonacademic biomedical career in that career area; (4) level of interest in a nonacademic biomedical career in workshop’s career area; (5) had the opportunity to explore different nonacademic biomedical career options in workshop’s career area; (6) have support in pursuing different career options at WSU. As with Phase I, when comparing biomedical target with non-biomedical departments, similar results for Phase II were obtained with a statistically significant pre-post change for each question (data not shown). In addition, between 91% and 97% of students in Phase I and Phase II seminars and workshops agreed or strongly agreed that the information provided was useful.

### Student participation in career sectors during Phase III (experiential learning)

Forty-four doctoral students in biomedical (70%) and non-biomedical (30%) departments participated in Phase III Experiential Learning programming, starting from an initial pilot phase in summer 2014 through fall 2017. BEST partnered with private companies, campus departments, local universities, community organizations, and municipal offices to provide trainees with relevant sites for their career explorations, typically during the late spring and summer months. The majority of awardees spent 15–20 hours a week for 6–8 weeks, although there was variability according to the schedule and commitments of the student and requirements of site supervising staff. Nine (20%) students reported spending up to 100 total hours, 24 (55%) reported 100–200 total hours, eight (18%) reported 240–400 total hours, and three (7%) reported over 400 hours on career exploration. A majority of opportunities were located in the Metro Detroit area or elsewhere in Michigan, but several were in other states and even in international settings. The most common career track was teaching at primarily undergraduate institutions (43% of the trainees who completed Phase III), followed by business and industry (32%). The remaining 25% of students had career explorations in other areas such as government regulation, science writing, and community engagement.

With the program being just in its fourth year, only a small number of BEST participants have graduated with their doctoral degrees (22 of 44 Phase III participants, as of December 2017). Of these 22 Phase III participant graduates, 4 chose to enter postdoctoral training (18%), 10 are pursuing careers in tracks in which they conducted their career exploration in Phase III (45%); and 6 are pursuing careers in a different track (27%) (the status of one graduated student is unknown, and one graduate is deceased). The remaining 21 Phase III awardees are still in training, and one entered medical school without completing the Ph.D. The 44 Phase III participants had virtually identical GPAs (average 3.73) and GREs (47.5 percentile), as shown in **Fig 3** for all BEST participants. The numbers in each career track are too small for comparison purposes.

## Discussion

Data collected from evaluation of the WSU BEST program revealed widespread interest among doctoral students in learning about careers beyond academia and the skillsets required to succeed across the spectrum of careers, reflecting national trends [9–15]. Also of note is the high interest across all demographics of students at WSU in learning about various career sectors. Similar to recent reports on interest in careers based on race and gender [17–27], we show robust participation from women and underrepresented students. It is important to note that over a three-year period, *almost half* of the students in biomedical departments participated in BEST program activities.

One of our goals was to determine if students with high GRE scores and doctoral GPAs participate in professional development activities at a rate different than those with lower scores, though we recognize that neither GRE nor GPA are the only measures of academic performance [28]. Nonetheless, our data show that at WSU there is no difference between the GREs and GPAs of students who participated in BEST activities (from 2014–17) compared to those who did not participate. Another goal was to determine if participation in professional development activities adversely impacts students’ completion of their training in a timely fashion. One of our key findings is that participation in such activities does not interfere with students’ abilities to perform their disciplinary training milestones and is not detrimental to their time-to-degree completion. If anything, based on our participation data (**Fig 1**), it can be argued that “low” to “moderate” amounts (“dosage”) of professional development activities are instead associated with faster degree completion times, suggesting that focused career planning by students may be more time efficient in securing a job than individual haphazard job searches. Additionally, since our programming is developed and delivered by our alumni and employers from these specific career sectors (and not just by academic faculty and administrators), students interact directly with practitioners in these careers. Students’ access to this large network of “career coaches” does not end when the BEST sessions are over. Many presenters spend additional time with students immediately after the conclusion of the BEST event or remain in contact with individual students in the long term, in some cases advising them in finding suitable jobs. Such mentorship opportunities can be invaluable to students’ future careers.

Our study results show that students are eager to learn about careers in a variety of sectors. In addition to academia (at research-intensive institutions), they are interested in the for-profit sector (business/industry), undergraduate teaching/PUIs, law/regulatory affairs, government, and communication. As our survey data reveal, participation in the three-phases of the BEST program led to self-perceived gains in knowledge among doctoral students about multiple career sectors, skills required for jobs in these sectors, and the ability to find resources to assist them in obtaining further information about careers. Importantly, by participating in these career exploration activities students were also able to rule out careers in which they were not interested (data not shown). Thus, we believe that the WSU BEST program empowers students to make informed decisions about the types of jobs to pursue after graduation and provides them with transferable skillsets to help them succeed in those paths, all essential for success in the training diaspora [9–11]. Access to networking with alumni and potential employers may also place participants at a strategic advantage in being hired for their first positions. Indeed, although our current data set is small, 72% of students who participated in Phase III Experiential Learning opportunities were able to find jobs in the career area that they explored in Phase III.

An important feature of the WSU BEST program in terms of sustainability is the “halo” effect it has created. At WSU, BEST activities are open to students from all programs. Doctoral students from a variety of departments (*e*.*g*., physics and astronomy, communication, and history) participate in BEST events, demonstrating the wide impact of our program beyond biomedical sciences. This inclusivity enhances cross-disciplinary interactions between students who otherwise may not have an opportunity to collaborate within the contexts of their doctoral research projects but who might work together in future career environments. These interactions enable them to appreciate different perspectives and engage in teamwork, a trait that employers seek. Inclusivity has been a hallmark of the WSU BEST program at all levels. BEST staff and steering committee members from disciplines as diverse as biomedical sciences, fine and communication arts, education, and social sciences work together to create programming. We believe that centralizing these activities, as well as the GPPDs, within the Graduate School will save faculty and individual programs’ resources and time. Recognizing the importance of this institution-wide delivery of programming has garnered long-term commitment and support from the administration, thus ensuring sustainability of the program.

While by no means unique to WSU, an overall challenge for programs to evaluate long-term impact is the lag from when students first start in the program to the time they complete training and begin their first job. Given an average of 5 years of doctoral and possible 3 years of additional postdoctoral training, the earliest career outcome might easily be 8 years past entering training. In other words, we cannot measure the impact of such programming on students for a minimum of 8 years at the very least. Thus, determining the long-term effects of the WSU BEST program and any corresponding programmatic changes will have to await this time period.

In summary, WSU BEST’s program has provided students with resources to explore careers in multiple sectors and to have the necessary skillsets to be successful in these careers. We found that doctoral students across all disciplines, and across gender, race, and citizenship status, participate equally in these activities. There was no association of GRE, GPA, or time-to-degree completion with students’ participation in these activities. Having programming available to all doctoral students, regardless of their discipline, has helped gain the acceptance and support of faculty (data not shown) as well as institutional financial commitment as they encourage students to explore careers beyond academia, leading to scalability and sustainability of our program at WSU. Our hope is for students and faculty alike from across the university to appreciate the centralized resources offered to trainees as they seek knowledge about careers across a wide array of sectors.

We believe that the data presented here demonstrate the keen desire of doctoral students to learn in a structured manner about the range of careers available to them. It underscores the desire of students, regardless of gender or race, to participate in these activities, and most importantly that participation in these career exploration and preparation activities do not adversely impact either academic achievements or time-to-degree completion.

## Supporting information

S1 File Survey instrument for Phase 1 module activities (example using business module). (PDF)

S2 File Survey instrument for Phase 2 module activities (example using business module). (PDF)
