# Supplementary material for: Exposure to multiple career pathways by biomedical doctoral students at a public research university
Source: PLoS One. 2018 Jun 22;13(6):e0199720. doi: 10.1371/journal.pone.0199720 (PMC6014666; doi:10.1371/journal.pone.0199720)
Supplement: S1 File — (PDF) [file pone.0199720.s001.pdf]

Business Survey: Phase 1 Module

Sponsored by the BEST Program

(REV. 08-05-15)

We value your opinion. We would greatly appreciate your evaluation of this seminar so we can improve future BEST offerings.

We'd like to know how much you knew about four areas BEFORE today's seminar (question #1 items) and AFTER today's seminar (question #2 items). Please respond to the following questions by circling a number, from 1 (nothing/not at all) to 5 (a great deal):

**1. *<u>Before</u>* attending today's seminar…**	Nothing/ Not at all ⟵			⟶	A great deal
a. how much did you know about nonacademic biomedical career options within business?	1	2	3	4	5
b. how much did you know about what skills are important for a nonacademic biomedical career in business?	1	2	3	4	5
c. how much did you know about the opportunities at Wayne State University to foster a nonacademic biomedical career in business?	1	2	3	4	5
d. how interested were you in a nonacademic biomedical career in business?	1	2	3	4	5

**2. *<u>After</u>* attending today's seminar…**	Nothing/ Not at all ⟵			⟶	A great deal
a. how much do you know about nonacademic biomedical career options within business?	1	2	3	4	5
b. how much do you know about what skills are important for a nonacademic biomedical career in business?	1	2	3	4	5
c. how much do you know about the opportunities at Wayne State University to foster a nonacademic biomedical career in business?	1	2	3	4	5
d. how interested are you in a nonacademic biomedical career in business?	1	2	3	4	5

Turn the page over >>>

Please respond to each of the following questions by circling a number, using the 5-point scale from 1 (strongly disagree) to 5 (strongly agree) and explain your rating:

		Strongly disagree	Disagree	Neutral	Agree	Strongly agree
3.	Overall, this seminar was worth attending.	1	2	3	4	5

Why/Why not?

		Strongly disagree	Disagree	Neutral	Agree	Strongly agree
4.	The seminar provided useful information.	1	2	3	4	5

Why/Why not?

5. Was there information you would have liked to have heard during this seminar that was not presented? If yes, please describe.

6. Was there information presented that you did not find useful? If yes, please describe.

7. Please share any comments or suggestions on other ways to improve this seminar.

8. Please check the types of ways you will take action from what you learned in this seminar. (Check all that apply)

☐A Share information with a faculty member (e.g. advisor, mentor, departmental colleagues)
☐B Share information with a peer
☐C Use strategies and/or information directly within my career development
☐D Pursue learning more about a nonacademic biomedical career in business
☐E Connect professionally with someone that I met
☐F None of the above
☐G Other next steps: (please describe) ___________________________________

9. Are you a: ☐1 Ph.D. student ☐4 postdoc
☐2 Masters student ☐5 faculty and staff
☐3 Undergraduate student ☐6 alumni

10. If you are a Ph.D. student, are you: ☐1 pre-candidacy ☐2 post-candidacy

11. Are you a student/postdoc at an institution *other than* Wayne State University: ☐1 Yes ☐2 No

12. Department/Program: ____________________________________

13. Is this the first BEST Phase 1 Module session you have participated in? ☐1 Yes ☐2 No ☐3 Unsure

Thank you for your feedback
