# Supplementary material for: Exposure to multiple career pathways by biomedical doctoral students at a public research university
Source: PLoS One. 2018 Jun 22;13(6):e0199720. doi: 10.1371/journal.pone.0199720 (PMC6014666; doi:10.1371/journal.pone.0199720)
Supplement: S2 File — (PDF) [file pone.0199720.s002.pdf]

Business Survey: Phase 2 Module

Sponsored by the BEST Program

(REV. 01-12-18)

We would appreciate your evaluation of this workshop so we can improve future BEST offerings.

1. Please tell us how certain you felt BEFORE attending any of BEST's Phase 1 one-hour seminars and NOW, after Phase 2...

BEFORE BEST's PHASE 1 ONE-HOUR SEMINARS (Nothing/Not at all → A great deal)						NOW (Nothing/Not at all → A great deal)				
1	2	3	4	5	a. How much did/do you know about nonacademic biomedical career options within business?	1	2	3	4	5
1	2	3	4	5	b. How much did/do you know about what skills are important for a nonacademic biomedical career in business?	1	2	3	4	5
1	2	3	4	5	c. How much did/do you know about the opportunities at Wayne State University to foster a nonacademic biomedical career in business?	1	2	3	4	5
1	2	3	4	5	d. How interested were/are you in a nonacademic biomedical career in business?	1	2	3	4	5
1	2	3	4	5	e. How much opportunity did/do you have to explore different nonacademic biomedical career options in business?	1	2	3	4	5
1	2	3	4	5	f. How much support in pursuing different career options did/do you have at Wayne State University?	1	2	3	4	5

2. Please tell us how certain you felt BEFORE attending this workshop and NOW, after the workshop...

BEFORE THIS BEST PHASE 2 WORKSHOP (Nothing/Not at all → A great deal)						NOW (Nothing/Not at all → A great deal)				
1	2	3	4	5	a. How much did/do you know about how products are conceptualized, designed, developed, and pitched within a corporate structure?	1	2	3	4	5
1	2	3	4	5	b. How much did/do you know about the elements of corporate culture?	1	2	3	4	5
1	2	3	4	5	c. How much did/do you know about the communication skills needed to be successful in different business environments?	1	2	3	4	5

Turn the page over >>>

3. What are the most important insights you had from this workshop?

4. Considering WSU's commitment to community engagement and its urban mission, did this seminar broaden your perception on how your knowledge may impact your community?
☐1 Yes ☐2 No

Please respond to each of the following questions by circling a number, using the 5-point scale from 1 (strongly disagree) to 5 (strongly agree) and explain your rating:

	Strongly disagree	Disagree	Neutral	Agree	Strongly agree
5. The workshop provided valuable information, extending my knowledge of business.	1	2	3	4	5

Why, or why not?

6. Was there information you would have liked to have heard during this workshop that was not presented? If yes, please describe.

7. Please share any comments or suggestions on ways to improve the content, format or venue (other than catering) of this workshop.

8. Please check how you will take action from what you learned in this workshop. (Check all that apply)
   - ☐A Share information with a faculty member (e.g. advisor, mentor, departmental colleagues)
   - ☐B Share information with a peer
   - ☐C Update my IDP
   - ☐D Pursue learning more about a biomedical career in business
   - ☐E Connect professionally with someone that I met
   - ☐F None of the above
   - ☐G Other next steps: (please describe) ____________________

9. Are you a: ☐1 Doctoral student ☐2 Masters student ☐3 Undergraduate student ☐4 Postdoc ☐5 Faculty and staff ☐6 Alum

10. If you are a doctoral student, are you: ☐1 pre-candidacy ☐2 post-candidacy

11. If you are a postdoc, how many years have you been a postdoc: ____ years

12. Institution you are with: ☐1 WSU ☐2 Other: ____________________

13. College: ____________________ Department: ____________________

14. If you are a student, what doctoral program are you in: ____________________

15. Is this the first BEST Phase 2 workshop you have participated in? ☐1 Yes ☐2 No ☐3 Unsure

Thank you for your feedback
